# Parathyroidectomy and the use of ioPTH. A survey of the United Italian Society of Endocrine Surgery (SIUEC)

**DOI:** 10.1007/s13304-025-02430-8

**Published:** 2025-10-22

**Authors:** Paolo Del Rio, Salvatore Sorrenti, Giovanni Docimo, Gabriele Materazzi, Mario Testini, Pietro Giorgio Calò, Marco Raffaelli, Maurizio Iacobone, Carmela De Crea, Eleonora Lori, Elena Bonati

**Affiliations:** 1 Unit of General Surgery, Department of Medicine and Surgery, University of Parma, Parma, Italy; 2 Department of Surgery, Sapienza University of Rome, Rome, Italy; 3 Division of Thyroid Surgery, University of Campania “L. Vanvitelli”, Naples, Italy; 4 Endocrine Surgery Unit, University Hospital of Pisa, Pisa, Italy; 5 Department of Precision and Regenerative Medicine and Ionian Area, University of Bari “Aldo Moro”, Bari, Italy; 6 Department of Surgical Sciences, University of Cagliari, Cagliari, Italy; 7 Research Center for Endocrine Gland and Obesity Surgery, Università Cattolica del Sacro Cuore, Rome, Italy; 8 Endocrine and Metabolic Surgery Unit, Fondazione Policlinico Universitario Agostino Gemelli IRCCS, Rome, Italy; 9 Endocrine Surgery Unit, Department of Surgery, Oncology and Gastroenterology, Padova University Hospital, Padua, Italy; 10 Endocrine Surgery Unit, Isola Tiberina Hospital, Gemelli Isola, Rome, Italy

**Keywords:** Primary Hyperparathyroidism, Endocrine surgery survey, Parathyroid surgery, Minimally invasive parathyroidectomy, Intraoperative PTH assay

## Abstract

Primary hyperparathyroidism (PHPT) is a frequent endocrine disorder, with surgery as the only curative option. Despite evolving guidelines, real-life management remains variable across centers. A national survey was conducted among members of the Italian Society of Endocrine Surgery (SIUEC) to evaluate current practices in PHPT surgery, including the use of minimally invasive approaches and intraoperative parathyroid hormone (ioPTH) monitoring. Eighty-eight endocrine surgeons participated. Minimally invasive parathyroidectomy was the preferred approach, especially among high-volume surgeons. ioPTH was used by 83% of respondents, although its necessity in cases with concordant imaging was debated. Cervical ultrasound and ^99m^Tc-sestamibi were routinely used for localization; choline PET/CT was employed selectively. Significant differences emerged between low- and high-volume surgeons in surgical technique, ioPTH timing, and use of neuromonitoring. The survey highlights heterogeneity in the management of PHPT in Italy, suggesting room for standardization and broader guideline adherence. Efforts are needed to promote uniform, evidence-based practices nationwide.

**Supplementary Information:**

The online version contains supplementary material available at 10.1007/s13304-025-02430-8.

## Background

Primary hyperparathyroidism (PHPT) is a frequent endocrine condition resulting from an overproduction of parathyroid hormone (PTH), which leads to elevated calcium levels in the blood [1]. In the vast majority of cases—around 80%—PHPT is due to a single parathyroid adenoma [2]. Less commonly, it may involve multiple glands, as in cases of hyperplasia or multiple adenomas. Parathyroid carcinoma represents a rare cause [3]. Surgery is the only curative option, as it allows for the complete removal of the hyperfunctioning tissue [4, 5]. However, the intraoperative challenge lies in identifying all hyperfunctioning glands, which can vary considerably in both number and anatomical location. Precise surgical exploration is therefore essential to achieve definitive cure and to minimize the risk of persistence or recurrence, which would require reoperation—often made more complex by scarring and distorted anatomy from prior surgery. Recent technological advances—including improved preoperative imaging, minimally invasive surgical techniques, and intraoperative parathyroid hormone (ioPTH) monitoring—have significantly enhanced the diagnostic and therapeutic management of PHPT, enabling more targeted interventions and better outcomes [4, 6–8]. These innovations have made it possible to shift from traditional bilateral neck exploration (BNE) to focused or minimally invasive parathyroidectomy (MIP) in well-selected patients [9, 10].

Despite these developments, the real-world management of PHPT remains highly heterogeneous [11, 12]. Notably, discrepancies in standards of care persist across centers, particularly between high- and low-volume surgeons, even though the condition is common and its treatment—especially in classical presentations—should be generally well standardized [13]. Furthermore, while multiple consensus documents and position statements on PHPT management exist, formal clinical guidelines remain few and often outdated [14–21]. Many of these documents are drafted primarily by endocrinologists, and tend to offer only limited discussion of surgical management, despite surgery being the sole curative option for PHPT.

In response to this gap, the United Italian Society of Endocrine Surgery (SIUEC) recently published recommendations to guide surgeons in the management of PHPT [18]. In the wake of this publication, we conducted a national survey targeting SIUEC members to capture a snapshot of current perioperative and intraoperative practices. Special emphasis was placed on the use of ioPTH monitoring and minimally invasive approaches, aiming to assess adherence to current recommendations and to map the landscape of parathyroid surgery in Italy. By analyzing responses across centers with varying surgical volumes, this study seeks to highlight prevailing trends, identify areas of inconsistency, and ultimately provide insight into how PHPT is currently treated in everyday clinical practice—where formal guidance is limited, and real-world experience often drives decision-making.

## Methods

### Survey design

A cross-sectional e-survey was developed using Google Forms to evaluate the current preoperative and intraoperative practices, including the use of ioPTH monitoring, in the surgical management of PHPT. The survey was disseminated via email to all members of SIUEC using the society's official mailing list. Responses were collected over three weeks in June 2024 by the national secretariat. The reporting of the survey results followed the CHERRIES (Checklist for Reporting Results of Internet E-Surveys) guidelines to ensure methodological rigor and transparency.

### Survey content

The questionnaire included 12 multiple-choice questions (Supp.1) designed to investigate various aspects of clinical practice, such as the annual surgical case volume, the imaging modalities used in preoperative assessments, and the surgical techniques employed. It also explored the application and timing of ioPTH monitoring, the interpretation of its results, and the use of intraoperative neuromonitoring (IONM). Participants were categorized into two groups based on their response to the first question: low-volume surgeons (< 20 parathyroidectomies per year) and medium-to-high-volume surgeons (≥ 20 parathyroidectomies per year). This classification was established according to volume thresholds reported in the literature [22, 23].

### Informed consent and ethical approval

Participants were informed about the study’s purpose, data storage protocols, and the voluntary nature of participation. Ethical approval was not required, as the study was based on voluntary participation in a survey with no intervention or sensitive data collection. All participants provided informed consent for data processing.

### Data protection and confidentiality

Survey data were collected using Google Forms, with responses stored in an Excel database. Measures to ensure confidentiality included secure access to the database and participant identification via unique email addresses.

### Survey administration

The e-survey, administered in Italian language, was closed, targeting only registered SIUEC members. An invitation link was provided through the society’s mailing list. Adaptive questioning was employed to reduce complexity and improve completion rates. Responses were analyzed only from fully completed questionnaires, ensuring data quality.

### Statistical analysis

No statistical corrections, such as weighting or propensity score adjustments, were deemed necessary due to the survey’s descriptive nature. Duplicate entries were identified and excluded by cross-referencing participant names and email addresses. A chi-square test was performed to assess differences between groups, with a significance level set at *p* = 0.050. The analyses were conducted using SPSS version 27.

## Results

**Fig. 1:**
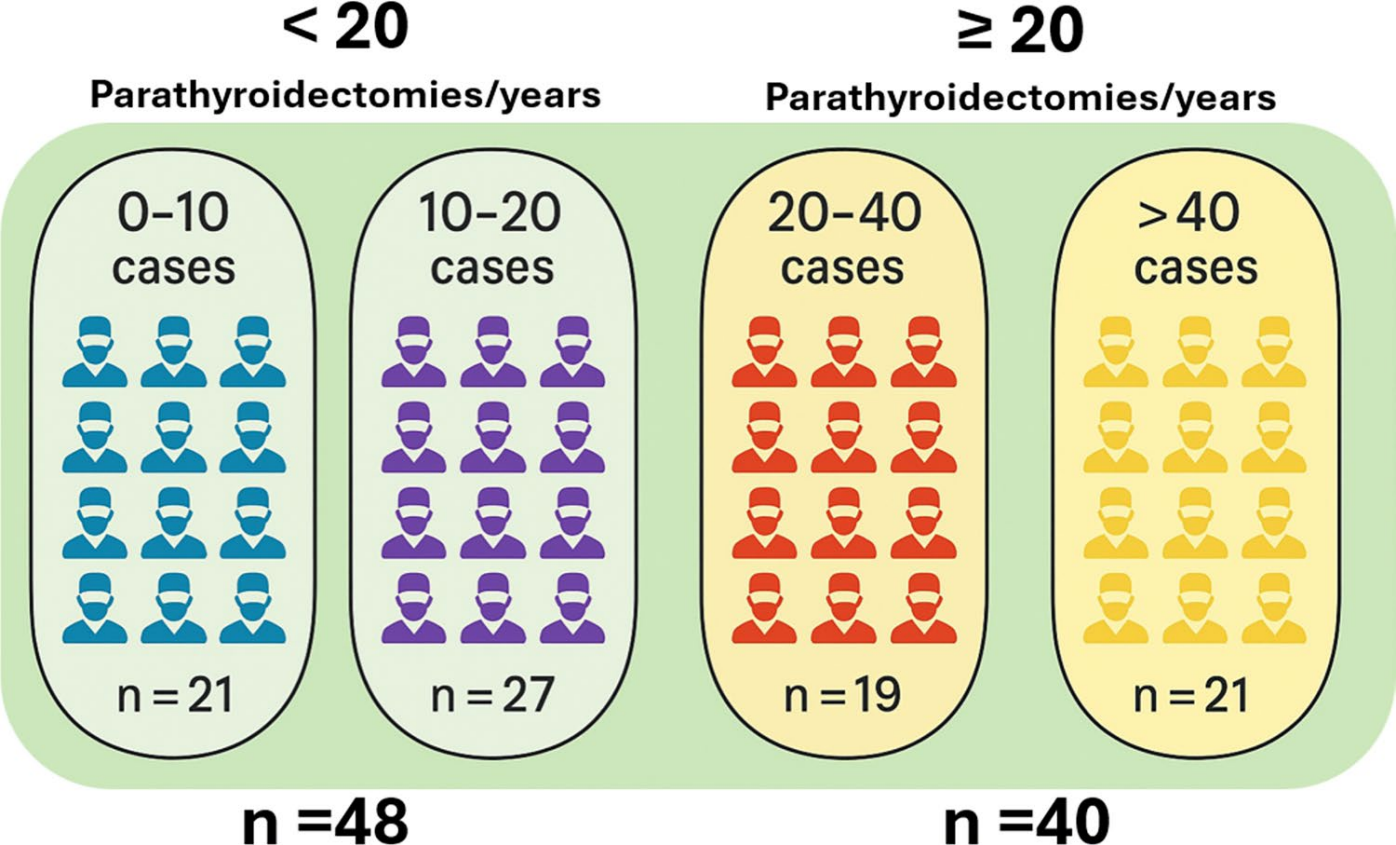
Surgeon case volume. Distribution of responders by annual number of parathyroidectomies performed, stratified by surgeon case volume (< 20 vs. ≥ 20 procedures/year)

A total of 88 SIUEC-member surgeons, evenly distributed across Italy, participated in the survey. Data analysis revealed that 21 surgeons performed fewer than 10 parathyroidectomies per year, 27 conducted 10–20 procedures annually, 19 performed 20–40 procedures, and 21 carried out more than 40 parathyroidectomies per year (Fig. 1).

**Fig. 2:**
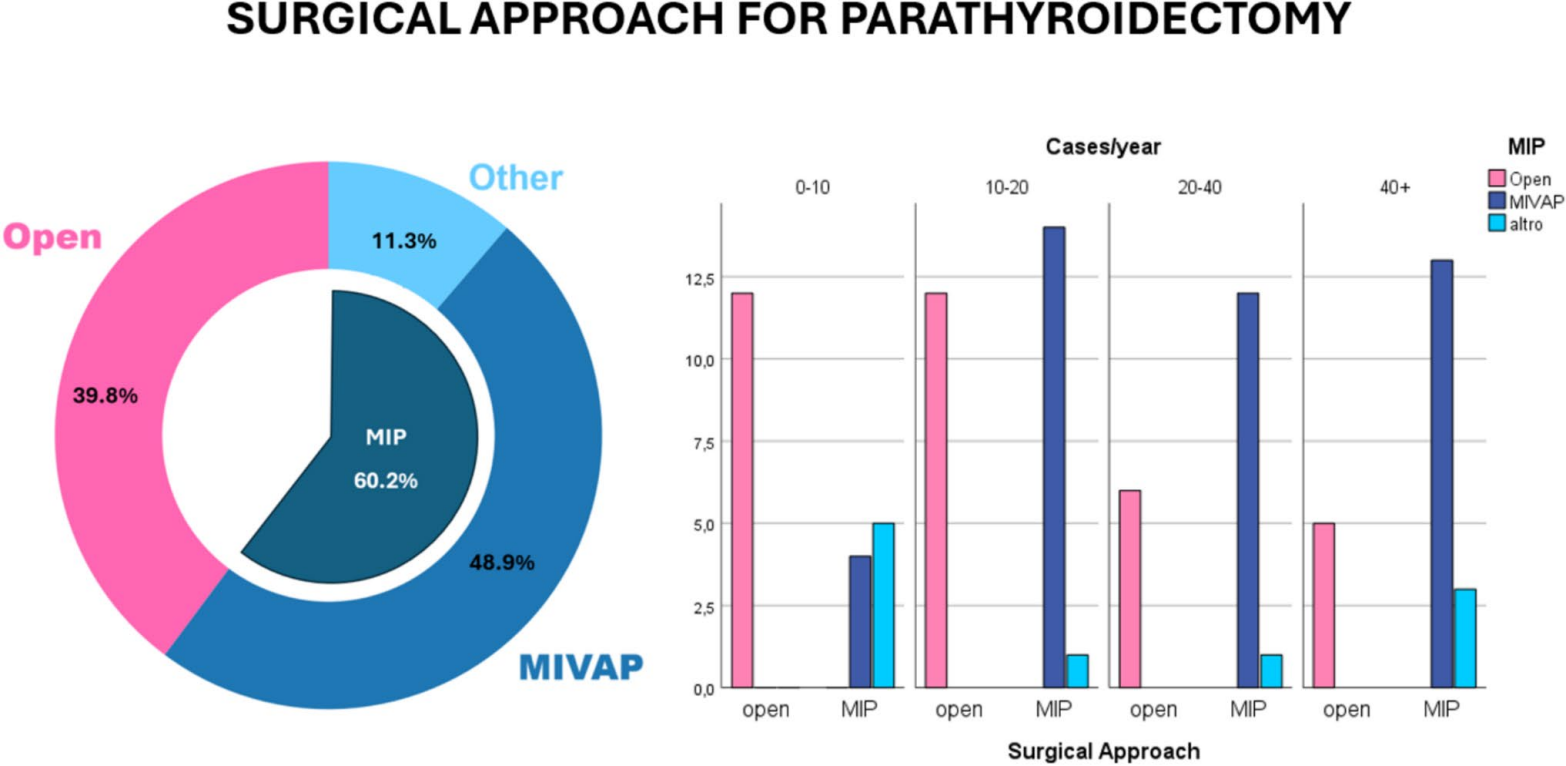
Surgical Approach for parathyroidectomy. Responses from surgeons regarding the choice of surgical approach for parathyroidectomy. On the left, a pie chart shows the overall distribution of responses, while on the right, a bar graph illustrates how different approaches are selected based on the surgeon’s activity volume

Minimally invasive surgery was identified as the primary approach for treating PHPT in 53 cases. Among these, the most frequently employed technique was minimally invasive video-assisted parathyroidectomy (MIVAP). A transaxillary approach was reported in only one case, while 11 surgeons adopted alternative techniques. Notably, no cases of transoral endoscopic parathyroidectomy video-assisted (TOEPVA) were reported (Fig. 2).

Regarding ioPTH testing, 73 out of 88 respondents indicated access to this technology. Of the 15 surgeons who did not use ioPTH, 12 performed fewer than 20 parathyroidectomies annually.

For preoperative imaging, 72 out of 88 respondents reported using cervical ultrasonography (US) combined with scintigraphy with ^99m^Tc-sestamibi (MIBI) and, when available, choline-PET. Six surgeons relied solely on US and MIBI, while three used US and ^18F^choline PET/CT. Notably, these three surgeons performed more than 20 parathyroidectomies per year.

Opinions on the necessity of ioPTH varied. Seventeen respondents deemed ioPTH unnecessary when US and MIBI scintigraphy results were concordant, while one respondent considered it superfluous when US and choline-PET findings aligned. These opinions were primarily expressed by high-volume surgeons. Conversely, 70 respondents advocated for the routine use of ioPTH.

**Fig. 3:**
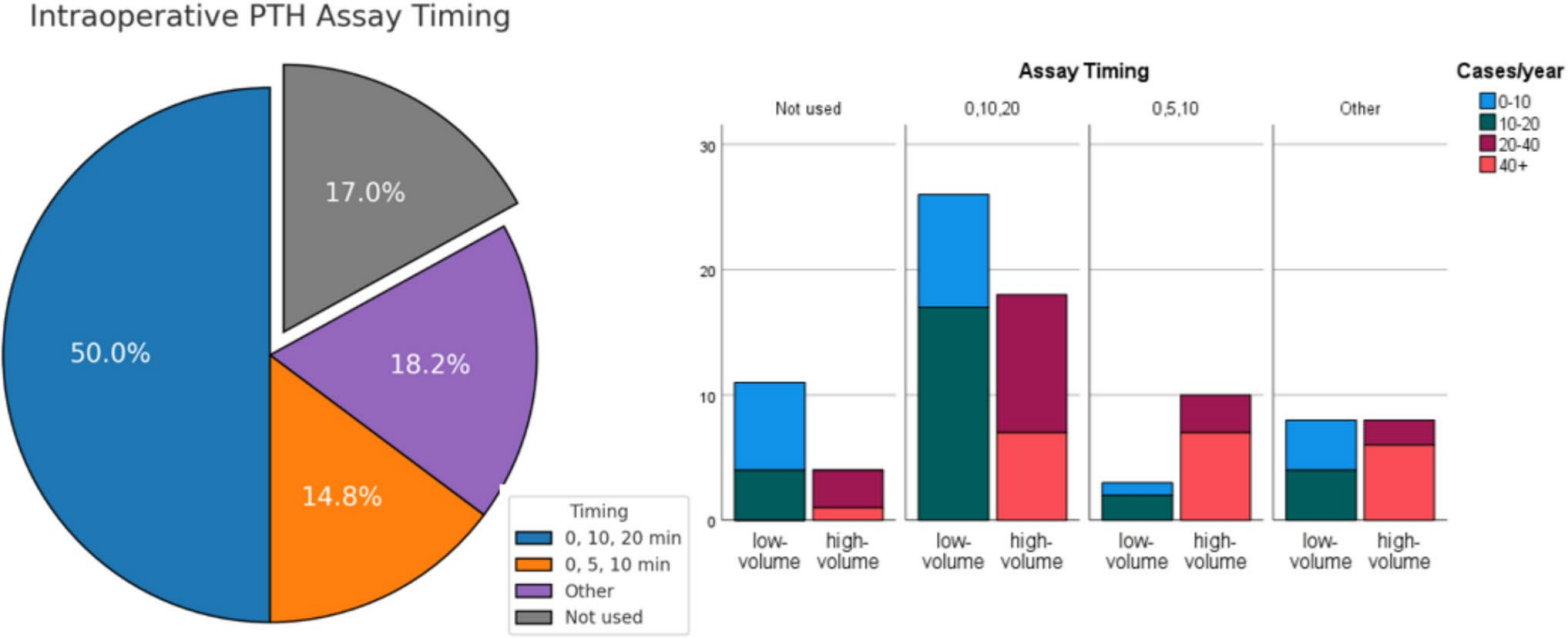
Intraoperative PTH Assay Timing. Distribution of intraoperative PTH assay timing preferences among endocrine surgeons and based on surgeon's activity volume

**Fig. 4:**
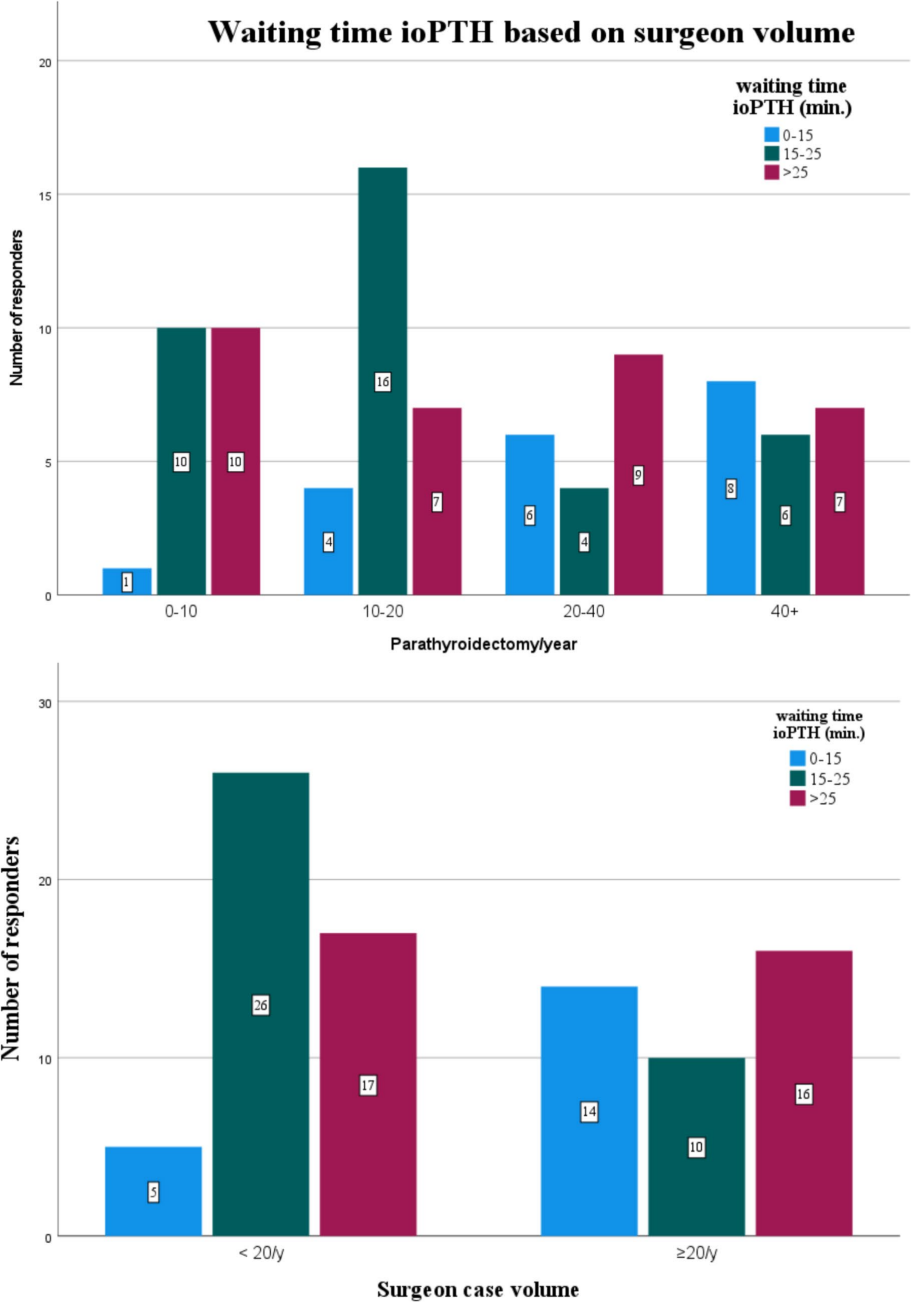
Waiting time for ioPTH. Panel A presents the waiting time categorized by the number of parathyroidectomies performed per year. Panel B groups the data by surgeon’s activity volume

Regarding the timing of the ioPTH measurement, 20 respondents reported a waiting period of less than 15 min, 36 suggested 15–25 min, and 32 preferred waiting more than 25 min (Fig. 3). A shorter waiting time was more frequently reported among surgeons performing at least 20 procedures annually (Fig. 4).

In cases where ioPTH levels failed to decrease adequately, 57 surgeons indicated a preference for bilateral cervical exploration, 10 ruled it out, and 21 considered it only in selected cases. The latter group primarily consisted of surgeons performing fewer than 20 procedures annually.

When conversion from a minimally invasive approach to cervical exploration was necessary, 58 respondents supported conversion to open surgery, with no significant differences observed across volume groups.

Fluorescence techniques for parathyroid gland identification were reported by 41 respondents, with usage evenly distributed across all volume categories.

**Fig. 5:**
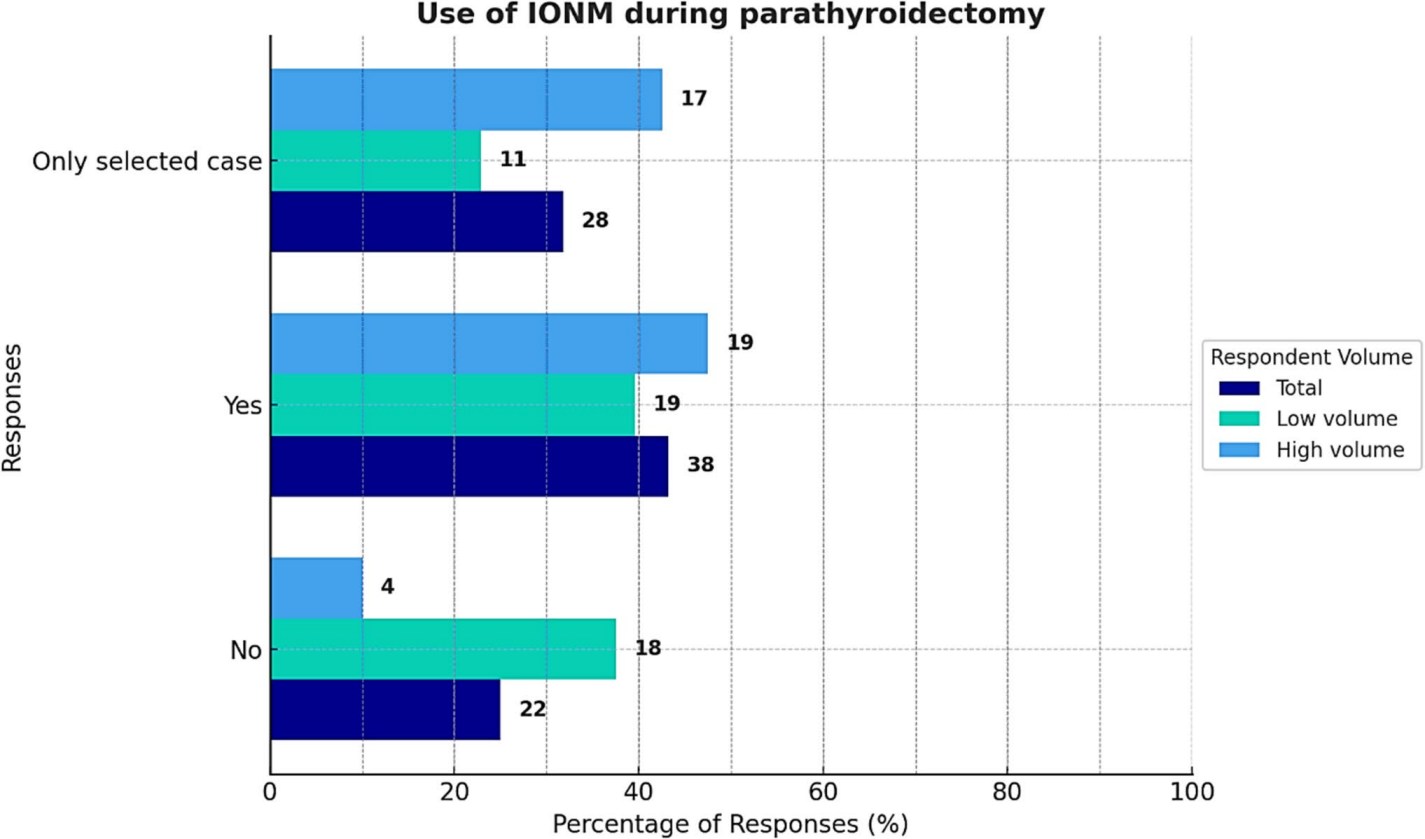
Use of intraoperative neuromonitoring (IONM) during parathyroidectomy based on the annual volume of parathyroidectomies performed by the surgeon

Finally, the routine use of IONM during parathyroidectomy was reported by 38 respondents. Selective use of IONM was indicated by 28 surgeons, while 22 reported never using it (Fig. 5).

For the statistical analysis, participants were categorized into two groups based on surgical volume: 54.5% (*n* = 48) performed fewer than 20 parathyroidectomies per year (low-volume group), while 45.5% (*n* = 40) performed 20 or more procedures annually (medium-to-high-volume group). The results are presented in Table 1.

**Table 1:** Survey results and statistical analysis

	Total 88 (100)	< 20 case/year 48 (54.5)	≥ 20 cases/year 40 (45.5)	*p*-value
**Approach**				**0.032**
Open	35 (39.8)	24 (50.0)	11 (27.5)	
MIP	53 (60.2)	24 (50.0)	29 (72.5)	
**MIP Approach**				0.058
MIVAP	43 (48.9)	18 (37.5)	25 (62.5)	
Other*	10 (11.3)	6 (12.5)	4 (10.0)	
**Use of ioPTH**				0.109
No	15 (17.0)	11 (22.9)	4 (10.0)	
Yes	73 (83.0)	37 (77.1)	36 (90.0)	
**Time for evaluation of ioPTH**				**0.050**
0, 10, 20 min	44 (50.0)	26 (54.2)	18 (45.0)	
0, 5, 10 min	13 (14.8)	3 (6.2)	10 (25.0)	
Other	16 (18.2)	8 (16.7)	8 (20.0)	
**Waiting for ioPTH results**				**0.005**
0–15 min	19 (21.6)	5 (10.4)	14 (35.0)	
15–25 min	36 (40.9)	26 (54.2)	10 (25.0)	
> 25 min	33 (37.5)	17 (35.4)	16 (40.0)	
**When ioPTH isn’t used**				0.247
Always use ioPTH	70 (79.6)	36 (75.0)	34 (85.0)	
Concordant imaging	18 (20.4)	12 (25.0)	6 (15.0)	
**In case of no descendent of ioPTH**				0.501
Never do a BNE	10 (11.3)	7 (14.6)	3 (7.5)	
Always do a BNE	55 (62.6)	30 (62.5)	25 (62.5)	
Sometimes do a BNE	23 (26.1)	11 (22.9)	12 (30.0)	
**Approach for a BNE**				0.392
Open	57 (64.8)	33 (68.7)	24 (60.0)	
MIP	31 (35.2)	15 (31.3)	16 (40.0)	
**Imaging**				0.213
US and MIBI	9 (10.2)	7 (14.6)	2 (5.0)	
US, MIBI and eventually PET-Choline	75 (85.2)	38 (79.2)	37 (92.5)	
US and PET-choline	4 (4.6)	3 (6.2)	1 (2.5)	
**Use of fluorescence technique**				0.413
No	46 (52.3)	27 (56.3)	19 (47.5)	
Yes	42 (47.7)	21 (43.7)	21 (52.5)	
**Use of IONM during parathyroidectomy**				**0.008**
No	22 (25.0)	18 (37.5)	4 (10.0)	
Yes	38 (43.2)	19 (39.6)	19 (47.5)	
Only selected cases	28 (31.8)	11 (22.9)	17 (42.5)	

All the results are expressed as number (percentage). A Chi-squared test was used to assess differences between groups and a p-value less than 0.050 was considered statistically significant

*MIP* minimally invasive parathyroidectomy, *MIVAP* Minimally invasive video-assisted parathyroidectomy, *ioPTH* intra-operative parathyroid hormone, *US* Ultrasound, *BNE* bilateral neck exploration

*Other approach are the remote access approach and robotic surgery

Regarding the surgical approach, MIP was more frequently performed than open surgery (53 vs. 35 cases). Notably, high-volume surgeons were significantly more likely to adopt MIP compared to low-volume surgeons (*p* = 0.032). Among those performing MIP, the most common approach was MIVAP (43 cases), with no statistically significant difference between groups (*p* = 0.058). Other approaches, such as robot-assisted surgery and remote access surgery, were little used.

The use of ioPTH monitoring was widespread, with 73 respondents (83%) reporting its use. No significant difference was observed between the two groups (*p* = 0.109). However, the timing of ioPTH assessment showed a statistically significant difference (*p* = 0.050), with low-volume surgeons favoring the 0, 10, and 20-min protocol (26 cases) compared to high-volume surgeons, who were more likely to use alternative timing strategies. Additionally, waiting time for ioPTH results significantly differed between groups (*p* = 0.005), with high-volume surgeons reporting a shorter waiting time (< 15 min in 35% of cases vs. 10.4% of cases in the low-volume group).

When ioPTH monitoring was not used, most respondents (*n* = 70, 79.6%) reported never relying solely on imaging concordance to proceed with surgery, with no significant difference between groups (*p* = 0.247). Similarly, there was no statistically significant difference in the surgical approach chosen to perform BNE in cases of inadequate ioPTH decrease (*p* = 0.501), with most respondents opting to always performing BNE. However, there were many responders that perform a BNE in case of a failure in ioPTH decrease, not always but only in selected cases (26.1%), with an equal distribution between the two groups.

In terms of imaging, the majority of participants relied on a combination of US and MIBI (75 cases, 85.2%) using choline-PET/CT in selected cases, while was less frequently used the association of US and choline-PET/CT instead of MIBI (9 cases). No significant difference was found between groups (*p* = 0.213).

The use of fluorescence techniques was reported by 42 respondents (47.7%), with no significant differences between the two groups (*p* = 0.413). Finally, the use of IONM during parathyroidectomy differed significantly between groups (*p* = 0.008). Low-volume surgeons were more likely to never use IONM (18 cases vs. 4), while high-volume surgeons reported a higher frequency of selective IONM use (17 cases vs. 11 in the low-volume group).

A summary of the survey results and the corresponding statistical analysis is presented in Table 1.

## Discussion

Recently, SIUEC published a paper outlining recommendations for the proper management of PHPT [18]. A recent review of the literature [19] shows that, despite the existence of multiple consensus statements and position papers, only a few formal clinical guidelines are currently available for PHPT management. This is likely due to the limited availability of robust evidence. Furthermore, most existing documents are outdated, with only a few being updated in the past five years. In light of this lack of clear and up-to-date guidelines, we sought to investigate how PHPT is actually managed in everyday clinical practice. Furthermore, many guidelines are developed by endocrinologists, and the surgical management of PHPT is often overlooked or given minimal attention, despite being the only definitive treatment for achieving a complete cure. This survey was distributed to all SIUEC members to assess adherence to current guidelines [18, 24] and best clinical practices, aiming to provide an overview of the real-world management of PHPT in Italy.

Data analysis revealed a heterogeneous distribution of surgical volumes among respondents, with a significant proportion of surgeons (54.5%) performing fewer than 20 parathyroidectomies per year. This finding suggests that, despite the increasing trend towards subspecialization, parathyroid surgery continues to be performed in low-volume centers and by less experienced surgeons.

Despite significant technological advances in recent years that have improved the management of PHPT—from diagnosis to surgical treatment—there are still notable discrepancies in standards of care across different centers [25]. These variations are especially evident when comparing high-volume surgeons to their low-volume counterparts [15–17, 22, 23]. This is surprising, given that PHPT is a common endocrine disorder and its treatment, particularly for classic cases, is generally well standardized [24, 26]. Comparative analysis between surgical and medical management in non-localized primary hyperparathyroidism emphasizes differences in renal and skeletal outcomes, providing indirect evidence on the clinical impact of different surgical strategies [27].

Considering that 80–90% of PHPT cases are due to a single adenoma located in the neck, preoperative imaging is crucial for optimal surgical planning, reducing operative time, and minimizing surgical extent [16, 24]. Each imaging modality has its advantages and limitations, and it is well recognized that different techniques offer complementary information. Thus, combining neck US, which enables evaluation of both the parathyroid and thyroid glands, with a nuclear medicine technique such as MIBI or, alternatively, choline PET/CT—an emerging tool for parathyroid localization—is currently recommended [16, 18, 28, 29]. In Italy, our survey shows that the preferred preoperative imaging strategy is the combination of cervical US and MIBI, with selective use of choline-PET/CT. This diagnostic approach is consistent with current international recommendations. Although choline-PET/CT has demonstrated superior diagnostic performance compared to MIBI, its use remains limited in Italy, mainly due to high costs, and is typically reserved for cases with non-concordant or inconclusive first-line imaging. Recent data from literature suggest that the choice of parathyroid localization techniques has evolved considerably over the past decade, highlighting the need for standardization of surgical approaches and ongoing collaboration between high-volume referral centers and peripheral hospitals [28–30].

Regarding the surgical approach, BNE remains the gold standard [16, 18]. However, recent consensus documents—such as the 2025 AFCE (Association Francophone de Chirurgie Endocrinienne) Consensus [14] and the 2021 Australian Position Statement [16]—recommend focused parathyroidectomy as the first-line approach in patients with single-gland disease confirmed by imaging. BNE is reserved for discordant imaging, confirmed multiglandular disease, or reoperations. Similarly, the 2024 SIUEC recommendations identify a focused approach—either open or video-assisted—as the first choice in patients with single-gland disease and concordant imaging [18]. Our survey data show a statistically significant difference between medium–high and low-volume centers, with high-volume surgeons showing a preference for minimally invasive or focused approaches. In contrast, surgeons performing fewer than 20 procedures per year showed no clear preference. Only a few centers reported the use of more innovative techniques, such as robotic surgery, suggesting limited dissemination of these methods in Italy.

The role of ioPTH monitoring remains debated: it is not necessary for BNE but becomes important in minimally invasive approaches, acting as a “biochemical histology” to confirm complete removal of hyperfunctioning tissue [31]. However, ioPTH alters surgical decision-making in only a small proportion of cases, especially when minimally invasive surgery is reserved for well-selected sporadic single-gland PHPT cases confirmed by two concordant imaging modalities [16]. Its cost-effectiveness is often questioned [32]. Therefore, ioPTH may be used selectively but is considered essential in focused approaches, especially when imaging is uncertain [18]. Recent evidence shows that ioPTH monitoring significantly enhances surgical accuracy and success, reducing the risk of persistent disease and supporting its selective use in patients undergoing focused parathyroidectomy [33]. In our survey, the use of ioPTH monitoring was reported in 83% of cases. Only a minority of surgeons (18 out of 88) deemed it unnecessary when imaging was concordant. Although no statistically significant difference was found (*p* = 0.247), low-volume surgeons were more likely to avoid ioPTH in cases of concordant imaging than high-volume surgeons (25% vs. 15%). This finding suggests that surgical experience may foster greater reliance on intraoperative biochemical confirmation than on preoperative imaging alone. Waiting times for ioPTH results differed significantly between surgical volume groups (*p* = 0.005), with high-volume surgeons more frequently reporting turnaround times of less than 15 min (35%). This may reflect the availability of dedicated ioPTH assay equipment in high-volume centers, whereas low-volume centers often rely on central laboratories, increasing both waiting and operative times.

In cases where ioPTH levels do not drop appropriately, the literature uniformly recommends proceeding with BNE, either open or video-assisted [9, 34, 35]. Despite this, only 57 out of 88 surgeons reported routinely performing BNE in such cases. A minority selectively proceed based on the clinical scenario, and 11.3% never perform BNE. However, no significant differences emerged between surgical volume groups, suggesting a uniform approach to suspected multiglandular disease when ioPTH fails to decrease. Most surgeons (64.8%) reported that BNE should be performed using an open technique, even when the initial approach was minimally invasive. Only a minority of surgeons—regardless of volume—chose to complete the operation via MIVAP, despite evidence supporting its safety in bilateral exploration [35].

Among adjunct intraoperative technologies to facilitate parathyroid identification, there is growing interest in fluorescence-based techniques—already widely used in thyroid surgery [36–38]. Although not yet standardized in parathyroid surgery, the use of indocyanine green (ICG) fluorescence and near-infrared autofluorescence (NIRAF) has proven helpful in locating parathyroid adenomas. Our survey found that 47.7% of respondents in Italy use fluorescence techniques intraoperatively, with no significant difference based on surgical volume. This suggests that although these methods are not yet standard, their growing use in thyroid surgery makes them accessible across all volume settings, thus promoting their adoption in parathyroid procedures.

The use of IONM for recurrent laryngeal nerves also remains controversial. While its utility is well recognized in complex cases and reoperations, routine use is not considered mandatory [14, 16, 18]. Unlike the French Consensus, which limits IONM to complex procedures, reoperations, and BNE, SIUEC has opted not to define specific indications. In our survey, IONM use showed a significant difference based on surgical volume: low-volume surgeons were less likely to use it (18 cases vs. 4 among high-volume surgeons). Conversely, high-volume centers more frequently used IONM selectively, likely reflecting greater access to the required equipment. Overall, IONM—whether used routinely or selectively—was reported by 75% of respondents, a figure that is increasing compared to previous reports, suggesting wider dissemination of this technology.

This study has several limitations, including the inherent bias of survey-based research due to reliance on self-reported data without external validation, the absence of clinical outcome measures, and the lack of detailed information on institutional resources that may influence surgical decision-making. The decision not to include questions on clinical outcomes was intentional, to avoid introducing an additional bias, as such questions could have led respondents to provide misleading information on either their adherence to guidelines or patient outcomes. Additionally, the exclusive participation of SIUEC members may introduce selection bias and limit the generalizability of the findings to the broader surgical community. SIUEC, as the national society of endocrine surgery, theoretically includes all Italian surgeons with a significant interest in this field. Nevertheless, it is difficult to estimate the number of surgeons with occasional involvement in endocrine surgery who were not reached by the survey. Despite this, SIUEC members represent the reference community for endocrine surgery in Italy, and their responses provide a comprehensive overview of current practice patterns among the most actively engaged surgeons.

In light of the differences observed in guideline adherence and of the recommendations themselves, future studies should address the need for a cost–benefit analysis of key aspects such as the use of intraoperative PTH monitoring, the routine employment of PET-Choline, and the standardized application of technologies for intraoperative parathyroid identification (e.g., indocyanine green, autofluorescence).

## Conclusion

In conclusion, this survey highlights substantial adherence to consolidated surgical practices. However, statistical analysis reveals differences in treatment standards between surgeons with varying operative volumes. The adoption of innovative techniques remains limited, while the use of novel diagnostic and intraoperative technologies varies according to surgical volume, likely due to cost constraints. These findings suggest the need to tailor training pathways by emphasizing exposure to high-volume centers, structured mentorship in advanced techniques, and standardized curricula that incorporate evolving technologies. Moreover, strategic planning at the institutional and national levels should aim to ensure equitable access to technological resources, support multidisciplinary collaboration, and foster centralized care models where appropriate. In particular, structured training in referral centers should provide all surgeons managing primary hyperparathyroidism with a comprehensive foundation, while the most complex cases should be referred to tertiary-level centers, where the greater surgical and overall experience of high-volume centers can optimize patient outcomes. These efforts may enhance the overall quality and consistency of parathyroid surgery in Italy.

## Supplementary Information

Below is the link to the electronic supplementary material.

Supplementary file1 (PDF 157 KB)

## Data Availability

The data in this article is derived from individual responses to the survey and is therefore confidential and not in the public domain.
