# Correction: Parathyroidectomy and the use of ioPTH. A survey of the united Italian society of endocrine surgery (SIUEC)

**DOI:** 10.1007/s13304-026-02568-z

**Published:** 2026-02-19

**Authors:** Paolo Del Rio, Salvatore Sorrenti, Giovanni Docimo, Gabriele Materazzi, Mario Testini, Pietro Giorgio Calò, Marco Raffaelli, Maurizio Iacobone, Carmela De Crea, Eleonora Lori, Elena Bonatipleaes, Elena Bonati, SIUEC PHPT Collaborative Group,

**Affiliations:** 1 Unit of General Surgery, Department of Medicine and Surgery, University of Parma, Parma, Italy; 2 Department of Surgery, Sapienza University of Rome, Rome, Italy; 3 Division of Thyroid Surgery, University of Campania “L. Vanvitelli”, Naples, Italy; 4 Gabriele Materazzi Endocrine Surgery Unit, University Hospital of Pisa, Pisa, Italy; 5 Department of Precision and Regenerative Medicine and Ionian Area, University of Bari “Aldo Moro”, Bari, Italy; 6 Department of Surgical Sciences, University of Cagliari, Cagliari, Italy; 7 Marco Raffaelli Research Center for Endocrine Gland and Obesity Surgery, Università Cattolica del Sacro Cuore, Rome, Italy; 8 Endocrine and Metabolic Surgery Unit, Fondazione Policlinico Universitario Agostino Gemelli IRCCS, Rome, Italy; 9 Maurizio Iacobone Endocrine Surgery Unit, Department of Surgery, Oncology and Gastroenterology, Padova University Hospital, Padua, Italy; 10 Research Center for Endocrine Gland and Obesity Surgery, Università Cattolica del Sacro Cuore, Rome, Italy; 11 Endocrine Surgery Unit, Isola Tiberina Hospital, Gemelli Isola, Rome, Italy

**Correction to: Updates in Surgery**

10.1007/s13304-025-02430-8

In this article, the order that the author Dr. Elena Bonati name appeared in the author list was incorrect. Author name to change in the right place and his institute name has been added.

The original article has been corrected.

