# Supplementary material for: Parathyroidectomy and the use of ioPTH. A survey of the United Italian Society of Endocrine Surgery (SIUEC)
Source: Updates Surg. 2025 Oct 22;78(1):351–63. doi: 10.1007/s13304-025-02430-8 (PMC12909413; doi:10.1007/s13304-025-02430-8)
Supplement: Supplementary file 1 — Supplementary file1 (PDF 157 KB) [file 13304_2025_2430_MOESM1_ESM.pdf]

Survey Questions – Italian/English

Cognome / *Surname*

Nome / *Name*

Indirizzo e-mail / *E-mail Address*

1) Quanti casi di iperparatiroidismo primitivo tratti/anno?
   How many primary hyperparathyroidism cases do you treat per year?
   □ 0-10
   □ 10-20
   □ 20-40
   □ > 40

2) Utilizzi tecnica tradizionale o mininvasiva/remota?
   Do you use traditional or minimally invasive/remote techniques?
   □ Si, MIP/ *Yes, MIP*
   □ No, open

3) Se hai risposto SI, quale tecnica prediligi?
   If you answered YES, which technique do you prefer?
   □ MIVAP
   □ Trans-ascellare / *Trans-axillary*
   □ Altro / *Other*

4) Hai la possibilità di eseguire il test rapido del PTH intraoperatorio (iPTH)?
   Do you have the possibility to perform the rapid intraoperative PTH test (iPTH)?
   □ Si / *Yes*
   □ No

5) Se hai risposto SI , che tempistica di prelievo applichi?
   If you answered YES, what timing do you apply for sampling?
   □ 0, 5, 10
   □ 0, 10, 20
   □ Altro / *Other*

6) Quale imaging utilizzi per la diagnosi di sede?
   Which imaging do you use for localization diagnosis?
   □ ecografia cervicale e PET con Colina / *Cervical ultrasound and PET with Choline*
   □ ecografia cervicale,scintigrafia con MIBI ed in caso dubbio PET con colina / *Cervical ultrasound, MIBI scintigraphy, and if in doubt, PET with choline*

◻ ecografia cervicale e scintigrafia con MIBI / *Cervical ultrasound and MIBI scintigraphy*

7) Quando decidi di non utilizzare il ioPTH?
When do you decide not to use ioPTH?
◻ Mai / *Never*
◻ Quando ecografia e PET con colina sono concordi / *When ultrasound and PET with choline are concordant*
◻ Quando ecografia e scintigrafia con MIBI sono concordi / *When ultrasound and MIBI scintigraphy are concordant*

8) Quanto tempo attendi per il risultato del ioPTH in media?
How long do you typically wait for the ioPTH result?
◻ >25 min
◻ 0-15 min
◻ 15-25 min

9) In caso di mancata caduta del ioPTH conduci un'esplorazione cervicale bilaterale?
In case of no ioPTH drop, do you perform bilateral cervical exploration?
◻ No
◻ A volte / *Sometimes*
◻ Sì / *Yes*

10) Se hai iniziato con tecnica mini-invasiva continui l'esplorazione bilaterale di principio con la stessa tecnica?
If you started with a minimally invasive technique, do you continue the bilateral exploration with the same technique?
◻ Si, sempre / *Yes, always*
◻ No, converto / *No, I convert*

11) Hai iniziato ad utilizzare tecniche di fluorescenza per l'identificazione delle paratiroidi?
Have you started using fluorescence techniques for parathyroid identification?
◻ Si / *Yes*
◻ no

12) Utilizzo l'IONM nella paratiroidectomia?
Do you use IONM during parathyroidectomy?
◻ Si / *Yes*
◻ No
◻ In casi selezionati / *In selected cases*
